# Pulmonary and systemic responses of highly pure and well-dispersed single-wall carbon nanotubes after intratracheal instillation in rats

**DOI:** 10.3109/08958378.2011.614968

**Published:** 2011-10-31

**Authors:** Norihiro Kobayashi, Masato Naya, Kohei Mizuno, Kazuhiro Yamamoto, Makoto Ema, Junko Nakanishi

**Affiliations:** 1 Research Institute of Science for Safety and Sustainability, National Institute of Advanced Industrial Science and Technology, Tsukuba, Japan; 2 National Metrology Institute of Japan, National Institute of Advanced Industrial Science and Technology, Tsukuba, Japan; 3 Research Institute of Instrumentation Frontier, National Institute of Advanced Industrial Science and Technology, Tsukuba, Japan

## Abstract

The present study was conducted to assess the pulmonary and systemic responses in rats after intratracheal instillation of highly pure, well-dispersed, and well-characterized SWCNTs. Exposure to SWCNTs up to 2mg/kg did not produce mortality, changes in clinical signs, or body weights during the observation period. Dose-dependent changes were observed in the lung weight, BALF inflammatory cells, and biochemical parameters such as LDH value, protein content, IL-1β and IL-6 activity, and histopathology. In the 0.04 mg/kg SWCNT-exposed group, almost no changes were observed during the observation period. In the 0.2 mg/kg SWCNT-exposed group, pulmonary inflammatory responses were observed after instillation. In the 1 mg/kg and 2 mg/kg SWCNT-exposed group, acute lung inflammation and subsequent granuloma accompanied by increased lung weights were observed. Furthermore, the histopathological findings in the lungs of rats exposed to SWCNTs showed inflammatory responses related with the vital reaction to the foreign substance that was instilled intratracheally, and there were no fibrosis, atypical lesion, or tumor-related findings even at the highest dose (2 mg/kg) of SWCNT-exposed groups up to 6 months after instillation. For all groups, histopathological changes due to the instillation exposure of SWCNTs were observed only in the lungs and lung-associated lymph nodes and not in the other tissues examined (i.e. the liver, kidney, spleen, and cerebrum).

## Introduction

Carbon nanotubes (CNTs) are fiber-shaped substances that consist of graphite hexagonal mesh planes (gra-phene sheets) in a single layer or as multiple layers with nest accumulation. Tubes with single-wall and multi-wall structures are termed single-wall carbon nanotubes (SWCNTs) and multi-wall carbon nanotubes (MWCNTs), respectively. CNTs are one of the most attractive nano-materials, because of their unique and excellent physico-chemical properties. Currently, various applied studies are focusing on CNTs.

However, there is growing concern regarding the hazards of CNTs. Many pulmonary toxicity studies (e.g., inhalation exposure studies [Shvedova et al., 2008a], intratracheal instillation studies [Chou et al., 2008; Lam et al., 2004; Miyawaki et al., 2008; Warheit et al., 2004], and pharyngeal aspiration studies [Mangum et al., 2006; Shevedova et al., 2005, 2007, 2008a, 2008b]) have reported that multifocal granulomas or fibrotic responses were persistently observed in rodent lungs after SWCNT exposure. MWCNT pulmonary toxicity studies also reported similar pulmonary responses as SWCNT exposure. Granulomatous inflammation and fibrotic responses were reported in MWCNT inhalation exposure studies (Li et al., 2007; Ma-Hock et al., 2009; Muller et al., 2005; Pauluhn et al., 2009).

Most of the previous CNT toxicity studies were conducted with agglomerated CNTs administrated to experimental animals (Chou et al., 2008; Lam et al., 2004; Li et al., 2007; Ma-Hock et al., 2009; Miyawaki et al., 2008; Morimoto et al., 2010; Muller et al., 2005; Mangum et al., 2006; Pauluhn et al., 2009; Shvedova et al., 2005, 2007, 2008a, 2008b; Warheit et al., 2004). However, some studies indicated that dispersed CNTs are more toxic than agglomerated CNTs when inhaled or instilled into the lungs of experimental animals (Mercer et al., 2008; Muller et al., 2005; Porter et al, 2010). Muller et al. (2005) reported that MWCNTs ground by a ball mill (average tube length: 0.7 μm) induced greater inflammation than non-ground bulk MWCNTs (average tube length: 5.9 μm) after intratracheal instillation in rats. Mercer et al. (2008) reported that after pharyngeal aspiration exposure of mice to dispersed SWCNTs (average particle size: 0.69 μm) and non-dispersed SWCNTs (average particle size: 15.2 μm), thickening of the alveolar walls was observed only in the group exposed to dispersed SWCNTs. Porter et al. (2010) suggested that dispersed MWCNTs could reach the pleura after pharyngeal aspiration exposure in mice. These findings indicate that toxicity studies using agglomerated CNTs may underestimate the hazards of CNTs. However, few toxicity studies have been performed on dispersed CNTs.

Furthermore, these studies were conducted using CNTs that contained substantial amounts of metal impurities. For example, SWCNTs containing as much as 18% (w/w) iron were assessed in the intratracheal instillation study by Shvedova et al. (2008a), and MWCNTs containing approximately 0.5 % of cobalt was assessed in an inhalation study by Pauluhn et al. (2009). Several investigators attributed some observed toxicity of CNTs to the metal impurities. To assess the pulmonary toxicity of CNTs, toxicity studies using CNT samples with low impurities are necessary.

The water-assisted chemical vapor deposition method (denoted as “super-growth CVD” method) produces very pure SWCNTs (99.98%), which are very desirable for electronic applications (e.g., in super capacitor, energy storage, sensing, etc.) and other potential industrial usages. Certain electronic applications required that CNTs be well-dispersed and free of metal contaminants; scientists and engineers have long been trying to purify and disperse CNTs. Therefore, we investigated highly pure, well-dispersed CNT in a preparation suitable for assessment of its toxicity. Measurements of pulmonary injury including histopathology, white blood cell counts, and biomarkers of oxidative stress and cytokine induction in bronchoalveolar lavage fluid (BALF) were conducted. Light microscopic and transmission electron microscopic examinations were also performed to evaluate translocation of SWCNTs in the lungs. Further, systemic responses of intratracheally instilled SWCNTs in rats were evaluated on the basis of histopathology.

Our study investigated the toxicity of highly pure and well-dispersed SWCNTs, which had not been investigated, and therefore, provides an assessment of toxicity of SWCNTs that is not confounded by the presence of metals, which likely contributed to some of the previous measures of the toxicity of SWCNTs.

## Materials and methods

### Animals

Seven-week-old male Crl: CD (SD) rats were purchased from Charles River Laboratories Japan, Inc. (Yokohama, Japan). The rats were kept in an animal facility and housed in positive-pressure air-conditioned units (19-25°C, 35-75% relative humidity) on a 12:12-h light/dark cycle. After 5 days acclimation, rats weighing 277-327 g were allocated to each group.

Animal experiments were performed in 2009 at the Kashima Laboratory, Mitsubishi Chemical Medience Corp. (Tokyo, Japan) in accordance with the Law for Partial Amendments to the Law Concerning the Protection and Control of Animals (2005).

This study was approved by the Institutional Animal Care and Use Committee of the Testing Facility and performed in accordance with the ethics criteria contained in the by laws of the Committee of the National Institute of Advanced Industrial Science and Technology.

### SWCNTs

SWCNTs were synthesized by the water-assisted chemical vapor deposition (super-growth CVD) method with iron as catalyst at the National Institute of Advanced Industrial Science and Technology, Japan. Super-growth CVD efficiently produces SWCNTs and the activity and lifetime of the catalysts are enhanced by addition of water vapor (Hata et al., 2004). SWCNTs synthesized using super-growth CVD have relatively large diameters (1-3nm), high carbon purity (above 99.98%), and high specific surface area (above 1000 m^2^/g). Super-growth SWCNTs are believed to be useful materials for various energy and material storage applications (Hiraoka et al., 2010).

### Preparation of SWCNT suspension

To disperse SWCNTs in liquid for intratracheal instillation, SWCNTs (0.04, 0.2, 1.0 or 2.0mg/mL) and 10mg/ mL of polyoxyethylenesorbitanmonooleate (Tween 80; MP Biomedicals LLC, CA, USA) were added to 10 mM of Phosphate Buffered Saline (PBS; EMD Biosciences, Inc., USA) dissolved in Milli-Q water (Millipore Corporation, Billerica, MA, USA).Samples were sonicated using an ultrasonic bath for 4h at 55 W and a frequency of 35 kHz. Temperature of the bath water was kept at 0-10 °C during sonication, because flocculation of SWCNTs occurs at higher temperatures. The above SWCNT suspensions were used for intratracheal instillation the day after preparation.

Tween 80 (10 mg/mL) in PBS (10 mM) was used as the negative (vehicle) control material. Min-U-Sil 5 crystalline silica particles (U.S. Silica Co., Berkeley Springs, *WV*, USA), which produce continuous pulmonary inflammation in the lungs of rats with 5 mg/kg of intratracheal instillation (Kobayashi et al., 2009, 2010; Warheit et al., 2004, 2007), were used as the positive control material. These negative and positive control materials were prepared by sonication as described for the SWCNT suspension. The concentration of the crystalline silica particles was adjusted to 5 mg/mL for intratracheal instillation.

### Characterization of SWCNTs

For the bulk SWCNT samples and SWCNT suspensions, tube morphology was evaluated on the basis of observations using a transmission electron microscope (TEM; JEM-1010; JEOL Ltd., Tokyo, Japan). Tube length and diameter of the SWCNT suspensions were measured with an atomic force microscope (AFM Dimension 3100 and Nanoscope IIIa controller,Veeco Instruments, Inc., USA). The Brunauer, Emmett, Teller (BET) specific surface area was measured by the N_2_ gas adsorption method (Belsorpmini II, Bel Japan, Inc., Osaka, Japan). Carbon impurities (i.e. amorphous carbon contents) and metal impurities of the bulk SWCNTs were measured by thermogravimetric analysis (TGA Q5000, TA Instruments, Inc., USA), where the samples were heated at a rate of 5°C/min in dry air atmosphere. Metal impurities were measured qualitatively and quantitatively by inductively coupled plasma-mass spectrometer (ICP-MS). Furthermore, the presence of defects in the graphene structure of the bulk SWCNTs and the SWCNT suspensions was evaluated by Raman spectroscopy (Nicolet Almega XR micro-Raman system, Thermo Fisher Scientific Inc., Japan). The resonance Raman scattering spectra were measured in the frequency regions of 100-3000 cm^-1^ with an excitation wavelength of 532 nm.

### Experimental design

**Figure 1:**
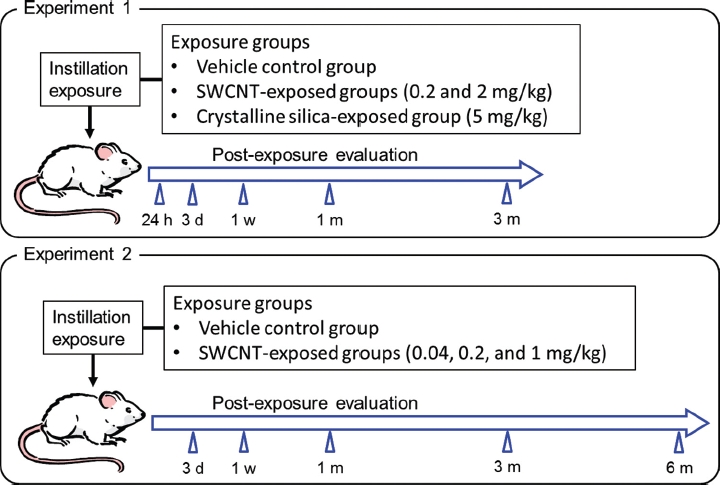
Summary of the experimental design of experiments 1 and 2.

Two intratracheal instillation experiments were conducted in the present study (see Figure 1). In the first experiment (experiment 1), pulmonary and systemic responses of SWCNTs were compared with those of crystalline silica up to 3 months after instillation. In the second experiment (experiment 2), we confirmed the reproducibility of the results of experiment 1. In addition, a detailed dose-response relationship and reversibility of the biological responses in rats intratracheally instilled with SWCNTs was examined up to 6 months after instillation.

In experiment 1, rats were anesthetized with ether, and 1 mL/kg body weight of 0.2 or 2.0 mg/mL of SWCNT suspension, 5 mg/mL of crystalline silica particle suspension (positive control), or Tween 80 solution (negative control) were instilled via the mouth into the trachea using a 18G fluorocarbon polymer cannula (#7204, Fuchigami Ltd., Kyoto, Japan). This corresponded to doses of 0.2 and 2 mg/kg body weight of SWCNTs and 5 mg/kg body weight of crystalline silica particles. Following instillation, the viability and general condition of the rats were observed once a day until dissection. The body weight of each rat was measured before instillation and once a week, until the animal was euthanized for assessment of toxicity. The right lungs of rats were lavaged for biomarker assessment; the left lungs (after weight recorded), livers, kidneys, spleens, and cerebrums of these rats were histopathologically evaluated at 24 h, 3 days, 1 week, 1 month (4 weeks), and 3 months (13 weeks) after instillation. Five rats per group were evaluated at each time point.

In experiment 2, rats were intratracheally instilled with 0.04, 0.2, or 1 mg/kg body weight of SWCNTs or vehicle control solution. Following instillation, the rats were examined at 3 days, 1 week, 1 month (4 weeks), 3 months (13 weeks) and 6 months (26 weeks) after instillation in a similar manner as experiment 1. Six rats per group were evaluated at each time point.

### Bronchoalveolar lavage

The rats were euthanized by administration of an intra-peritoneal injection of pentobarbital sodium (Nembutal injectable, Dainippon Sumitomo Pharma Co., Ltd., Tokyo, Japan) followed by exsanguination. The left bronchus was clamped with forceps and the right bronchus was cannulated. Subsequently, 3mL of heated (37 °C) saline (Otsuka Pharmaceutical Factory, Inc., Tokushima, Japan) was filled and aspirated to and from the lung to recover the first BALF fraction (approximately 2 mL). The supernatant was obtained by centrifuging the BALF at 300 × g for 5 min and was used for biochemical and cytokine measurements. Thereafter, 2 mL of saline solution was filled and aspirated to and from the lung twice, and additional BALF (approximately 4 mL) was obtained, centrifuged at 300 × g for 5 min after addition to the precipitation obtained by centrifugation of the first BALF. The cell fraction was used to determine cell counts in the BALF.

### BALF inflammatory cell counts

The cell fractions were suspended in saline with the addition of BSA (0.1%) and EDTA-2K (0.05 mM) dissolved in PBS. The number of total cells and percentages of neutrophils, macrophages, lymphocytes, and eosinophils were counted with an automatic erythrocyte analyzer (XT-2000iV, Sysmex Corporation, Hyogo, Japan).

### BALF biomarkers measurements

Lactate dehydrogenase (LDH) and total protein (TP) concentrations in the supernatant obtained by centrifugation of the BALF were measured with an automatic biochemical analyzer (TBA-200FR, Toshiba Medical Systems Corporation, Tochigi, Japan). Interleukin (IL)-1α, IL-1β, IL-2, IL-4, IL-6, IL-10, granulocyte monocyte colony stimulating factor (GM-CSF), interferon (IFN)-γ, and tumor necrosis factor (TNF)-α concentrations were measured using a Rat Cytokine 10-Plex A Panel kit and Bio-Plex Suspension Array System (Bio-Rad Laboratories; Inc., Hercules, CA, USA).

The cell fractions were also analyzed for hemeoxygenase (HO)-1mRNA expression with TaqMan® One-Step RT-PCR Master Mix Reagent Kits and ABI PRISM® 7700 Sequence Detection System (Applied Biosystems, Carlsbad, CA, USA).

### Histopathological evaluation

The trachea, left lung, liver, kidney, spleen, and cerebrum were fixed with 10% (v/v) neutral phosphate-buffered formalin solution, embedded in paraffin, sectioned, and stained with hematoxylin and eosin (H&E) for histopathological evaluation under the light microscope. The histopathological evaluation was performed in a blind fashion. Further, the results were peer reviewed by another certified veterinary pathologist. Diagnostic criteria for histopathological evaluation were identical to those of our previous study (Kobayashi et al., 2009; 2010).

### Processing of lung tissue for transmission electron microscope (TEM)

The right lung from one rat per group from each time point was prepared for transmission electron microscopy examination. The lung tissues were fixed using glutaraldehyde and osmium tetroxide solution, dehydrated in ethanol, and embedded in epoxy resin. The specimens were stained with a 2% uranyl acetate solution and 0.5% lead citrate solution at room temperature. Conventional TEM observation was performed within an H-7000 (Hitachi, Japan) at an acceleration voltage of 80 kV. High-resolution observation was performed by an energy-filtering TEM method using an EM 922 (Carl Zeiss SMT, Germany) equipped with an OMEGA energy filter. Zero-loss filtering, which can increase the scattering and phase contrast of the TEM image, was carried out for the non-stained specimens.

### Statistical analyses

Each of the experimental values, with the exception of histopathological findings, was compared to its corresponding control in each time point. Statistical significance was determined using multiple comparison tests between the negative control and SWCNT-exposed groups. First, the Bartlett's test was conducted. One-way layout analysis of variance was conducted when the variances were equal. Dunnett's multiple comparison tests were conducted when the differences between the groups were significant. The Kruskal-Wallis test was used when the variances were not equal and Steel's multiple comparison tests were conducted when the differences were significant. Statistical significance was determined between the positive and negative control groups using intergroup comparison tests. First, the F-test was conducted; the Student's *t*-test was used when the variances were equal, and the Aspin-Welch *t*-test was used when the variances were not equal. Statistical significances were judged at the 0.05 probability level. SAS System version 6.12 (SAS Institute Japan Ltd., Tokyo, Japan) was used for all statistical analyses.

## Results

### Characterization of SWCNTs

**Figure 2:**
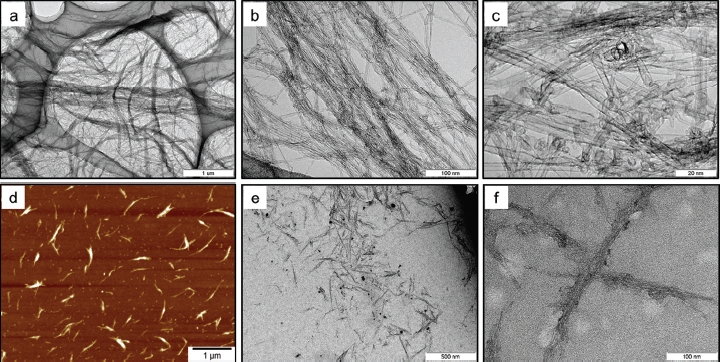
Morphology of the bulk SWCNTs and SWCNTs dispersed in the testing solution. Panels a-c showtransmission electron microscopy (TEM) images of bulk SWCNTs at different magnifications. Panel d shows an atomic electron microscopy (AFM) image of SWCNTs dispersed in the testing solution. Panels e and f show TEM images of SWCNTs dispersed in the solution at different magnifications.

Fundamental characteristics of the bulk SWCNTs and the dispersed SWCNTs in the testing solution are summarized in Table 1. TEM and AFM images of the bulk SWCNTs and the dispersed SWCNTs are presented in Figure 2. Based on the TEM observations, all of the CNTs contained in the bulk samples were present as single-wall, and other types of CNTs, such as double-wall and MWCNTs, were not observed (Figures 2a-c). The diameter of the nano-tubes was measured to be 3.0 ± 1.1 nm (mean ± SD). Total metal content, amorphous carbon content, and specific surface area were analyzed in 10 samples collected from different parts of the bulk material in order to evaluate homogeneity. Total metal content was estimated to be 0.05 ± 0.16 % (w/w, mean ± SD) by means of measurement of ash content (i.e. noncombustible remains) in TGA. This result demonstrated high purity of the bulk SWCNT material with little incorporation of metal particles that were used in the manufacturing process. TEM observation and quantification by ICP-MS supported this result. Amorphous carbon impurities are believed to be oxidized at lower temperatures than carbon nanotubes (NIST, 2008). The amorphous carbon content was estimated to be less than 2.3 ± 0.56% (mean ± SD) from the weight loss at 350°C, because SWCNTs of the bulk sample began to be oxidized at ∼500°C in the TGA. Specific surface area was determined to be 1064 ± 37 m^2^/g (mean ± SD) by BET method.

**Table 1:** Characterization of bulk SWCNTs and SWCNTs dispersed in the testing solution.

Sample	Characteristic	Value	Measuring method
Bulk SWCNTs	Tube diameter	3.0 ± 1.1nm^a^	Transmission electron microscopy (TEM)
	Maximum bundle length	1200 μm	Micrometer
	BET surface area	1064 ± 37m^2^/g^a^	N_2_ gas adsorption
	D/G ratio	0.14	Raman spectroscopy
	Amorphous carbon content	< 2.3 ± 0.56 %^a^	Thermogravimetric analysis (TGA)
	Total metal content	0.05 ± 0.16%^a^	
	Each metal content		Inductively coupled plasma- Mass spectrometry (ICP-MS)
	Fe	145 ppm	
	Ni	103 ppm	
	Cr	34 ppm	
	Mn	2 ppm	
	Al	12 ppm	
SWCNTs dispersed in the testing solution	Bundle diameter	12.0 ± 6.5 nm^a^	Atomic force microscopy (AFM)
	Bundle length	0.32 μm(1.76)^b^	
	D/G ratio	0.19	Raman spectroscopy
	pH	7.2	pH meter

a Values are expressed as mean ± SD.

b Values are expressed as geometric mean (geometric standard deviation).

In the testing solutions, SWCNTs were present in bundled forms due to their strong van der Waals interaction (Vaisman et al., 2006). In the present study, SWCNTs were suspended in bundled form with each bundle consisting of several SWCNT single fibers (Figures 2d-f). The diameter and length of the SWCNT bundles were measured from digital images acquired by AFM (Figure 2d). After a measurement of 120 bundles from 10 images, the diameter was determined to be 12 ± 6.5nm(mean ± SD), and the length was 0.32 μm (1.76) (geometric mean—geometric standard deviation), respectively (Table 3). The SWCNT length in the present study was shorter than those reported in Warheit et al. (2004) (> 1 μm), but similar to those reported in Shvedova et al. (2008a) (100-1000 nm). In the present study, SWCNTs were easily cut into these lengths when dispersed into the solution by ultrasonication. Therefore, the ultrasonication time produces no significant difference in the SWCNT length in suspension. The resonance Raman scattering spectra of the bulk SWCNT samples and the 0.2 and 2 mg/mL SWCNT dispersions are shown in Figure 4. Generally, intense sonication processes to achieve homogenous dispersion of SWCNTs into vehicles can cause degradation in SWCNT quality by introducing defects in the crystalline structure of SWCNT and breaking it into carbon debris. In order to evaluate the SWCNT quality, an effective method is to calculate the ratio of the intensities of disorder-induced mode (D-band) and grapheme-induced mode (G-band), which appears in the Raman spectrum. The variation of the D/G ratio is ascribed to the change in structural disorder on CNT surfaces (Dillon et al., 2005; Dresselhaus et al., 2005; Lee et al., 2008; Musumeci et al., 2008). In the present study, the D/G ratios of the bulk material and the dispersion solution were calculated to be 0.14 and 0.19, respectively, suggesting that there was only a slight drop in SWCNT quality of the dispersion solution.

**Figure 3:**
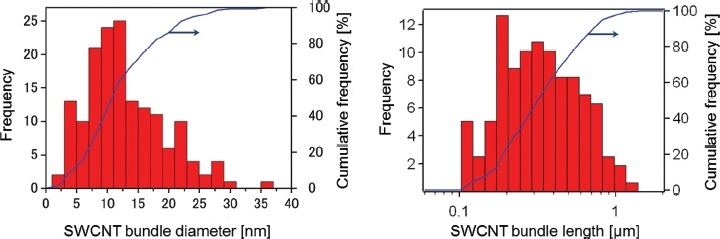
Distribution of SWCNT bundle diameter (left) and length (right) in 2 mg/mL SWCNT dispersion measured from digital images acquired by atomic force microscopy.

**Figure 4:**
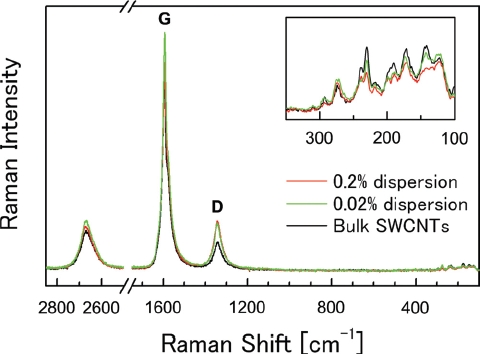
The resonance Raman scattering spectra of bulk SWCNT samples, and 0.2 and 2 mg/mL of SWCNT dispersions in the frequency regions of 100-3000 cm^4^. The inset shows the spectra of the low frequency region. Abbreviations G and D denote G-band and D-band, respectively.

**Table 3:** Summary of the results in experiments 1 and 2.

Endpoint		SWCNT-exposed group				Silica- exposed group
	Mass [mg/kg]	0.04	0.2	1.0	2.0	5.0
Dose	Surface area [m^2^/kg]	0.04	0.2	1.0	2.0	0.025
	Number [particle/kg]	1.8 × 10^12^	8.8 × 10^12^	4.4 × 10^13^	8.8 × 10^13^	7.3 × 10^8^
Clinical sign		-	-	-	-	-
Body weight		-	-	-	-	-
Lung weight		-	+	++	+++	-
	Inflammatory cells	-	+	++	++++	+++
BALF	LDH/protein	-	+	++	++++	+++
	IL-1β	-	+	++	+++	-
Histopathology	Pulmonary inflammation	-	+	++	++++	+++
	Inflammation (other tissues)	-	-	-	-	-

Note: Within the table, ++++ indicates the greatest change, where + indicates the least change. - indicates no significant change compared to the corresponding control.

### General condition and body and lung weight

SWCNT-related clinical signs of toxicity (e.g., abnormal behavior, irregular respiration, and piloerection) were not found in any groups of rats during the observation period in both experiments 1 and 2. (Table 3)

Statistically significant differences in the body weights of experimental animals were not observed between any of the SWCNT or crystalline silica-exposed groups or the control group during the experimental period in experiment 1 and 2.

**Figure 5:**
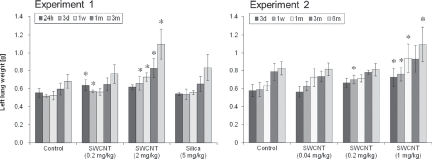
Absolute left lung weights of SWCNT-exposed rats and corresponding controls at each time point in experiment 1 (left) and 2 (right). Values are represented as the mean ± SD. *Significant increase from vehicle control (*p* < 0.05).

Lung weight was significantly increased in the 1 mg/ kg and 2mg/kg SWCNT-exposed groups compared to the control group until 3 or 6 months after instillation and in the 0.2mg/kg SWCNT-exposed group until 3 days or 1 week after instillation (Figure 5). There was no significant difference in lung weight between the 0.04mg/ kg SWCNT-exposed group and control group (Figure 5). The lung weight of the 5mg/kg crystalline silica-exposed group was not significantly increased in the present study, although a significant increase in lung weight was observed in the 5mg/kg crystalline silica-exposed group in our previous study at 6 months after instillation (Kobayashi et al., 2010). Relative lung weight (compared to body weight) showed the same tendency as the absolute lung weight.

### Necropsy findings

No abnormality was found at any of the time points in the vehicle control group and the 0.04mg/kg SWCNT-exposed group. In the 0.2mg/kg and higher dose of SWCNT-exposed groups, black spots were observed in the lung until 3 or 6 months after instillation. These spots were considered to be the pigments of the agglomerated SWCNTs in the lung. In all the groups, the black spots were not found in the other organs (i.e. the liver, kidney, spleen, and cerebrum) at any of the time points.

In the crystalline silica-exposed group, significant changes were not observed until 1 month after instillation; however, white patches were observed in the lung at 3 months after instillation, and enlargement of the right peritracheobronchial and parathymic lymph nodes were also observed.

### BALF inflammatory cells

**Figure 6:**
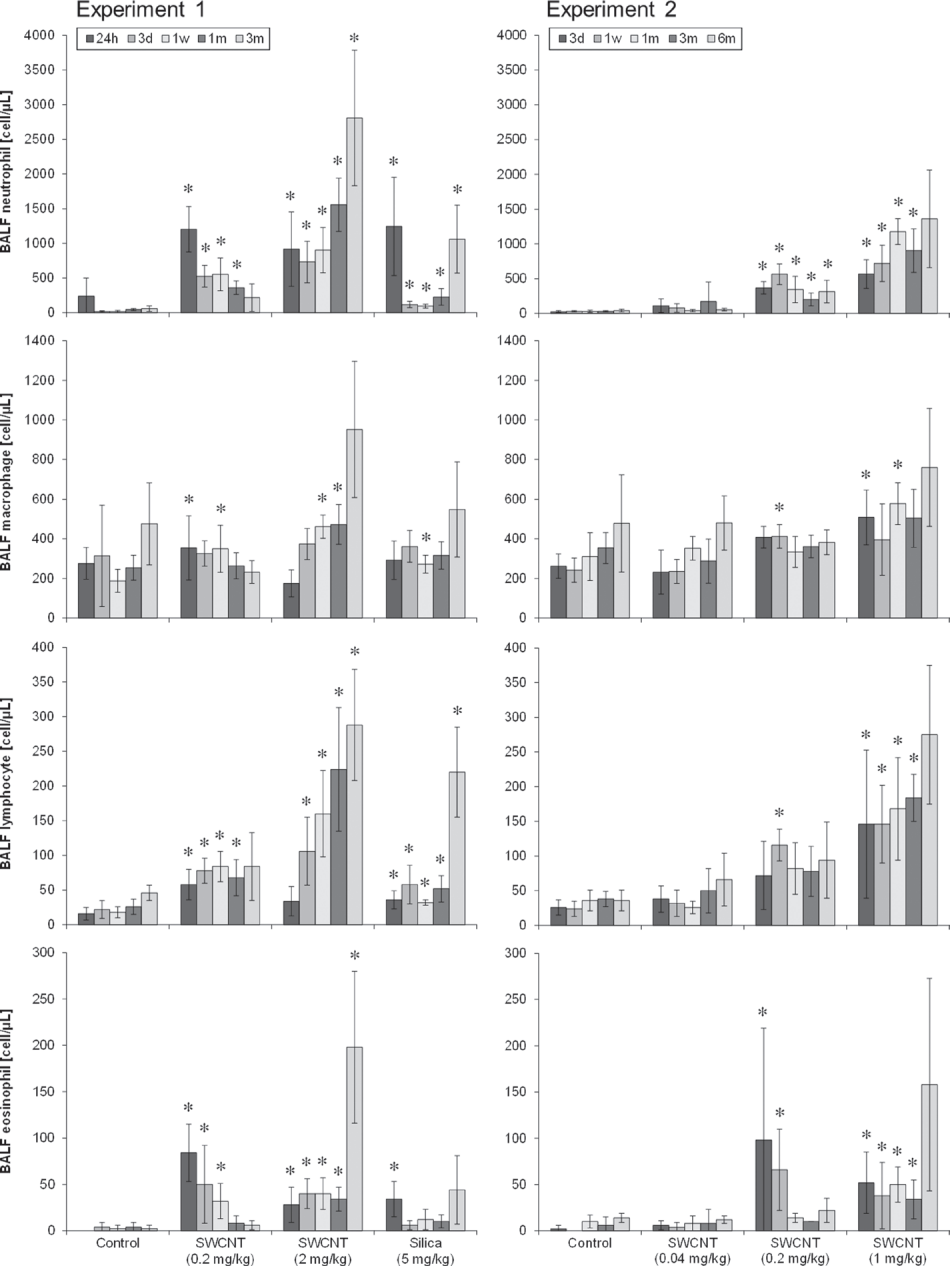
Number of neutrophils (a), macrophages (b), lymphocytes (c), and eosinophils (d) in BALF of SWCNT-exposed rats and corresponding controls at each time point in experiments 1 (left column) and 2 (right column). Values are represented as the mean ± SD. *Significant increase from vehicle control (p< 0.05).

In the SWCNT-exposed groups, the number of BALF inflammatory cells were increased in a dose-dependent manner in experiments 1 and 2 (Figure 6). In the 0.04 mg/kg SWCNT-exposed group (experiment 2), almost no changes were observed in BALF inflammatory cells. In the 0.2 mg/kg SWCNT-exposed group (experiments 1 and 2), BALF inflammatory cells increased after instillation, and some of them were recovered 3 or 6 months after instillation. In the 1 mg/kg (experiment 2) and 2 mg/kg (experiment 1) SWCNT-exposed groups, all the inflammatory cells in BALF significantly increased during the observation period. In the crystalline silica-exposed group, all the inflammatory cells in BALF significantly increased up to 3 months after instillation. The responses in the crystalline silica-exposed group were consistent with those observed in our previous studies (Kobayashi et al., 2009, 2010).

### BALF biomarkers

**Figure 7:**
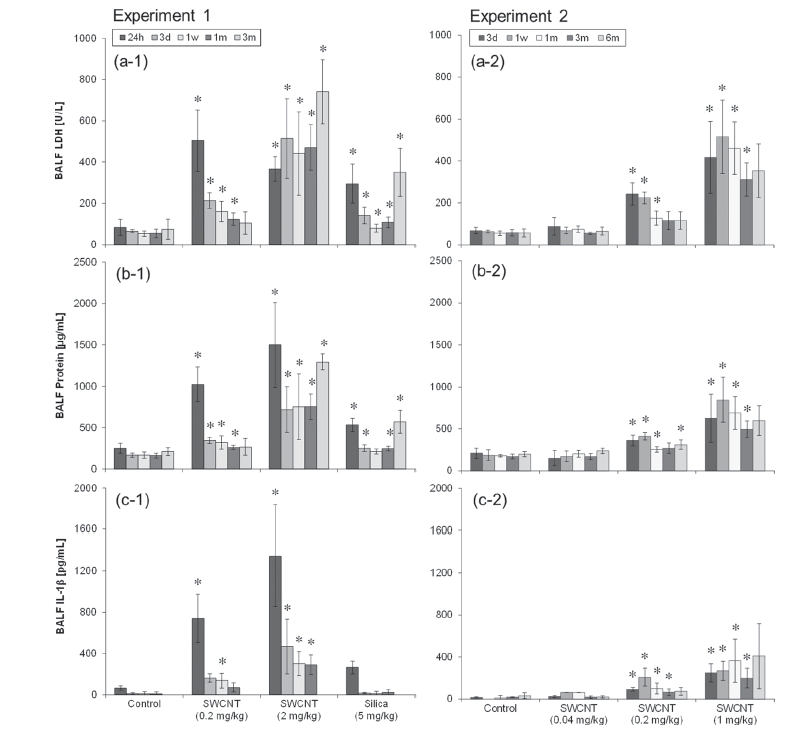
LDH value (a), protein content (b), and IL- Inactivity (c) in BALF of SWCNT-exposed rats and corresponding controls at each time point in experiments 1 (left column) and 2 (right column). Values are represented as the mean ± SD. *Significant increase from vehicle control (*p* < 0.05).

LDH values and protein contents in BALF were significantly greater in the 0.2 mg/kg and higher dose of SWCNT-exposed groups compared with those in the control group up to 3 months after instillation (Figures 7a and 7b). No significant changes were observed at any of the time points in the 0.04mg/kg SWCNT-exposed group.

Regarding the cytokine measurements, only small differences were observed in IL-1α, IL-2, IL-4, IL-10, GM-CSF, INF-γ, or TNF-α (data not shown) between the SWCNT or crystalline silica-exposed groups and the control group at any of the time points. Significant increases were observed only in IL-1β and IL-6 at several time points. IL-1β activity increased in the 0.2 mg/kg, 1mg/ kg, and 2 mg/kg SWCNT-exposed groups up to 3 months after instillation (Figure 7c). In the 0.04mg/kg and crystalline silica-exposed group, no significant changes were observed at any of the time points. IL-6 activity increased only in the 0.2 and 2.0 mg/kg SWCNT-exposed group at 24 h after instillation in experiment 1 (data not shown).

There was no significant difference in the relative amounts of HO-1 mRNA in BALF, between SWCNT or crystalline silica-exposed groups and the control group at any of the time points (data not shown).

### Histopathological evaluation

**Figure 8:**
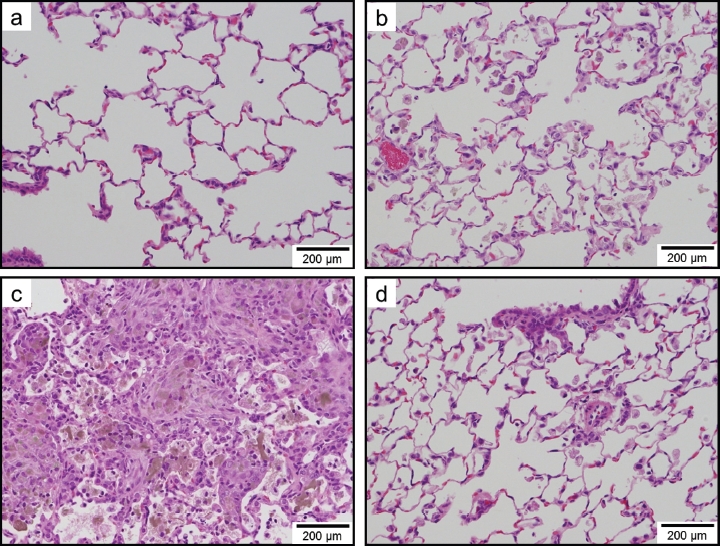
Light micrographs of lung tissue sections of rats at lweek after instillation in experiment 1 (H&E stain). No significant changes were observed in the vehicle control group (panel a). Minimal macrophage accumulation was observed in the alveoli of the 0.2 mg/kg SWCNT-exposed group (panel b). Moderate macrophage accumulation accompanied with foamy macrophages and mild inflammatory cell infiltration in the alveoli, and moderate macrophage infiltration and granuloma in the interstitium were observed in the 2.0 mg/kg SWCNT-exposed group (panel c). Minimal macrophage accumulation was observed in the alveoli of the crystalline silica-exposed group (panel d).

**Figure 9:**
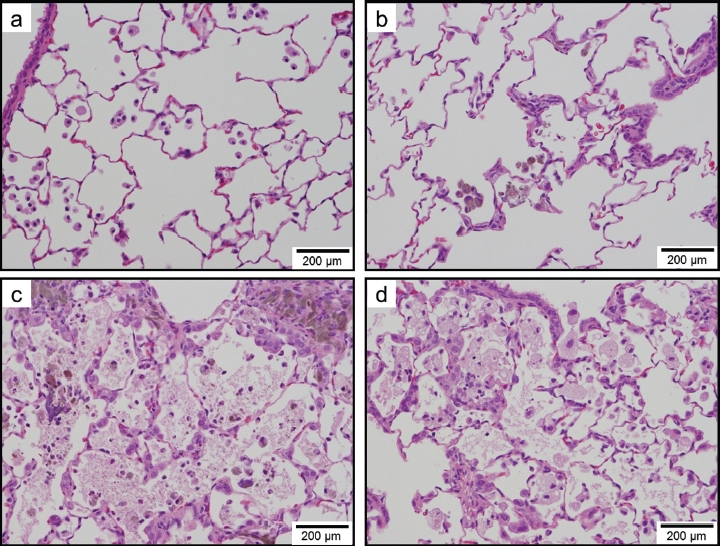
Light micrographs of lung tissue sections of rats at 3 months after instillation in experiment 1 (H&E stain). No significant changes were observed in the vehicle control group (panel a). Minimal macrophage accumulation was observed in the alveoli of the 0.2 mg/kg SWCNT-exposed group (panel b). Moderate macrophage accumulation accompanied with foamy macrophages, mild inflammatory cell infiltration in the alveoli, mild macrophage infiltration and granuloma in the interstitium, and mild hypertrophy of alveolar epithelium were observed in the 2.0mg/kg SWCNT-exposed group (panel c). Mild macrophage accumulation accompanied with foamy macrophages, minimal inflammatory cell infiltration in the alveoli, and minimal hypertrophy of alveolar epithelium were observed in the crystalline silica-exposed group (panel d).

**Figure 10:**
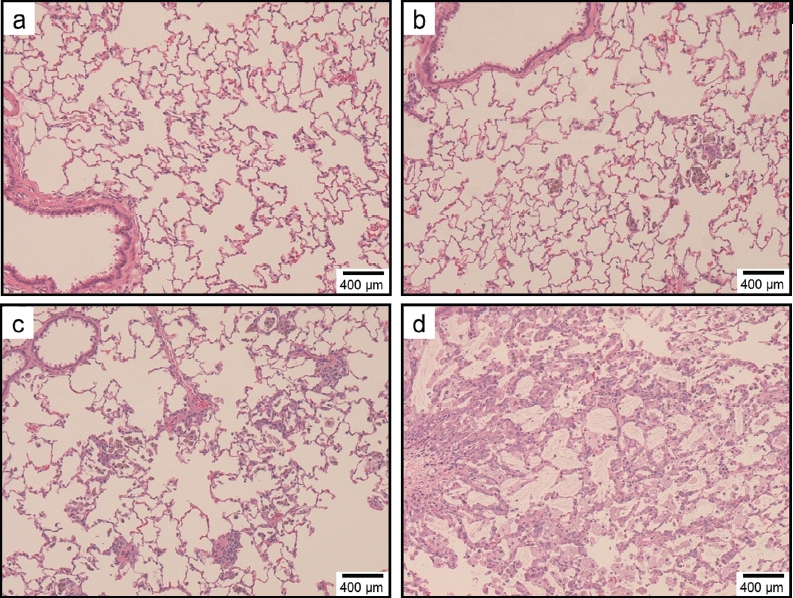
Light micrographs of lung tissue sections of rats at 6 months after instillation in experiment 2 (H&E stain). Minimal macrophage accumulation was observed in the alveoli of the vehicle control group (panel a). Minimal macrophage accumulation was observed in the alveoli of the 0.04 mg/kg SWCNT-exposed group (panel b). Minimal macrophage accumulation in the alveoli and interstitium were observed in the 0.2 mg/kg SWCNT-exposed group (panel c). Mild macrophage accumulation accompanied with foamy macrophages and minimal inflammatory cell infiltration, minimal macrophage infiltration in the interstitium, and minimal hypertrophy of alveolar epithelium were observed in the 1 mg/kg SWCNT-exposed group (panel d).

Table 2 summarizes the histopathological findings of the rats and their severity scores at each time point in experiments 1 and 2. Light micrographs of lung tissue sections of rats at 1week (experiment 1), 3 months (experiment 1), and 6 months (experiment 2) after instillation are presented in Figures 8-10, respectively. For all groups, histopathological changes due to the instillation exposure of SWCNTs or crystalline silica were observed only in the lungs and lung-associated lymph nodes, and not in the other tissues examined (i.e. the liver, kidney, spleen, and cerebrum). The histopathological findings of the lungs and lymph nodes in experiments 1 and 2 are detailed below.

**Table 2:** Pulmonary histopathology severity scoresM* of rats in experiments 1 and 2.

			SWCNT-exposed group				
				
Findings	Time point	Vehicle control group	0.04 mg/kg	0.2 mg/kg	1.0 mg/kg	2.0 mg/kg	Silica-exposed group
Administered substance accumulation, alveolus	24 h	0	–	1.0	–	3.0	0
	3 d	0	1.0	1.2	2.0	2.6	0
	1 w	0	1.0	1.0	1.4	2.4	0
	1 m	0	0.8	1.0	1.6	1.8	0
	3 m	0	0.8	1.1	1.6	2.0	0
	6 m	0	0.8	1.0	1.5	–	–
Administered substance accumulation, interstitium	24 h	0	–	0	–	1.8	1.8
	3 d	0	0	0.1	1.4	0	0
	1 m	0	0	0.1	1.4	1.2	0
	1 m	0	0	0.1	0.6	1.2	0
	3 m	0	0	0.1	1.4	2.0	1.0
	6 m	0	0	0.8	1.0		–
Inflammatory cell infiltration, alveolus	24 h	1.6	–	1.6	–	1.8	1.8
	3 d	0.1	1.0	0.6	1.2	0	0
	1 w	0	0	0.4	0.4	1.2	0
	1 m	0.2	0	0.2	0.8	1.2	0
	3 m	0	0	0.3	0.2	2.0	1.0
	6 m	0	0	0	0.8	–	–
Macrophage accumulation, alveolus	24 h	1.4	–	1.4	–	1.0	0
	3 d	0.4	1.0	1.2	2.0	0.8	0.2
	1 w	0.1	0.8	1.0	1.4	2.0	0.4
	1 m	0.1	0.8	1.1	1.8	1.6	0
	3 m	0.2	0.8	1.0	2.0	1.8	0.5
	6 m	0	0.8	1.0	1.8	–	–
Foamy macrophage, alveolus	24 h	0	–	0	–	1.0	0
	3 d	0	0	0	0	0.8	0.2
	1 w	0	0	0.1	0	2.0	0.4
	1 m	0	0	0.1	0	1.6	0
	3 m	0	0	0.1	1.6	1.8	0.5
	6 m	0	0	0	1.3	–	–
Macrophage accumulation, interstitium	24 h	0	–	0	–	0	0
	3 d	0.2	0	0.3	1.8	1.6	0
	1 w	0.1	0.2	0.3	1.0	2.4	0
	1 m	0	0	0.1	0.6	1.6	0
	3 m	0	0	0.1	1.4	1.8	0.5
	6 m	0	0	0.8	1.0	–	–
Granuloma	24 h	0	–	0	–	0	0
	3 d	0.2	0.2	0.3	2.0	1.4	0
	1 w	0	0	0	1.0	2.2	0
	1 m	0	0	0	0.4	0.8	0
	3 m	0	0	0	0.6	1.6	0.3
	6 m	0	0	0	0	–	–
Foreign body giant cell	24 h	0	–	0	–	0	0
	3 d	0	0	0	0	0	0
	1 w	0	0	0	0	0	0
	1 m	0	0	0	0	0.6	0
	3 m	0	0	0	0	0.8	0
	6 m	0	0	0	0	–	–
Hypertrophy of alveolar epithelium	24 h	0	–	0	–	0	0
	3 d	0	0	0	0	1.6	0
	1 w	0	0	0	0	1.6	0
	1 m	0	0	0	0	1.0	0
	3 m	0	0	0	0	2.0	0.8
	6 m	0	0	0	0	–	–
Hypertrophy of bronchial epithelium	24 h	0	–	0	–	1.0	0
	3 d	0	0	0	0	0.8	0
	1 w	0	0	0	0	1.6	0
	1 m	0	0	0	0	0	0
	3 m	0	0	0.1	1.0	0.8	0
	6 m	0	0	0	0.5	–	–
Alveolar proteinosis	24 h	0	–	0	–	0	0
	3 d	0	0	0	0	0.6	0
	1 w	0	0	0	0	1.4	0
	1 m	0	0	0	0	1.8	0
	3 m	0	0	0	0	2.4	1.3
	6 m	0	0	0	0	–	–

* Severity scores given to individual animals from a complete pathological examination are 0, not remarkable; 1, minimal; 2, slight/mild; 3, moderate; and 4, severe; based upon relative evaluation of lesions. Severity scores for each animal within a group (5 or 6 rats) were added, and an average score per animal was calculated, which is shown in the table.

– Not evaluated.

In the control group, minimal macrophage accumulation and minimal inflammatory cell infiltration in the alveoli was observed only at 24 h (experiment 1) and 3 days (experiment 2) after instillation (Figures 8a, 9a and 10a). These pulmonary inflammations were considered an artifact due to the instillation of 1 mL/kg liquid into rat lungs.

In the 0.04mg/kg SWCNT-exposed group, pulmonary responses were similar to the control group (Figure 9b). Minimal alveolar macrophage accumulation was observed up to 6 months after instillation, and inflammatory cell infiltration was observed only at 3 days after instillation in the 0.04mg/kg SWCNT-exposed group.

In the 0.2 mg/kg SWCNT-exposed group, macrophage accumulation in the alveoli and interstitium were observed up to 6 months after instillation (Figures 8b, 9b, and 10c). Inflammatory cell infiltration in the alveoli was observed up to 3 months, but not at 6 months after instillation. The pulmonary responses of rats exposed to 0.2 mg/kg SWCNTs were very similar between experiments 1 and 2.

In the 1 mg/kg or 2 mg/kg SWCNT-exposed group, the grade of the pulmonary inflammation was more severe than the 0.2 mg/kg SWCNT-exposed group (Figures 8c, 9c, and 10d). In addition to the histopathological findings observed in the 0.2mg/kg SWCNT-exposed group, foamy alveolar macrophages, hypertrophy of alveolar and bron-choalveolar epithelium, granuloma, and foreign body giant cells were persistently observed. In addition, pulmonary proteinosis seems to be induced in these groups on the basis of light microscopic observation (Figures 9d and 10d). In the 1 mg/kg or 2 mg/kg SWCNT-exposed group, reversibility of the pulmonary inflammation was not observed during the observation period in the present study.

In the crystalline silica-exposed group, accumulation of alveolar macrophages accompanying foamy macrophage in the alveoli, macrophage infiltration in the interstitium, inflammatory cell infiltration in the alveoli, hypertrophy of alveolar epithelium, and granuloma were persistently observed (Figures 8d and 9d). Reversibility of the pulmonary inflammation was not observed during the observation period in the present study.

The severity of the inflammatory responses evaluated based on histopathology was consistent with that of BALF inflammatory cells and biochemical measurements.

### Transmission electron microscopy (TEM) observation of SWCNTs deposited in the lungs

**Figure 11:**
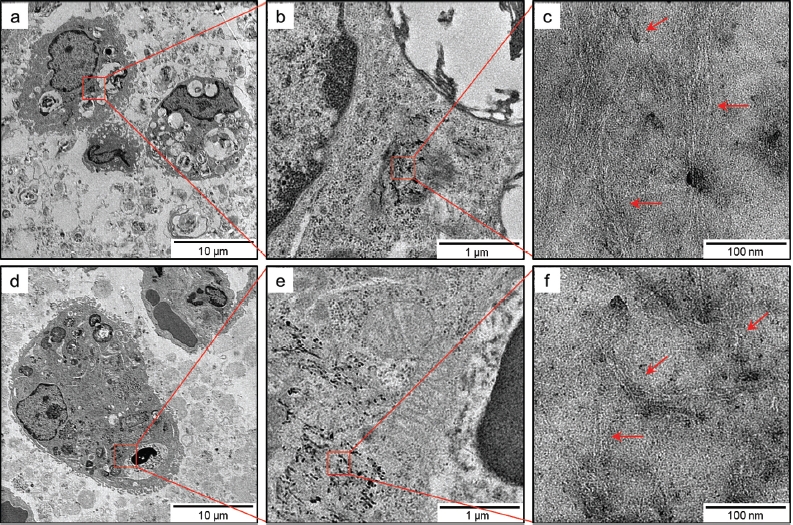
TEM images of SWCNTs deposited in lungs of rats exposed to 2.0 mg/kg SWCNTs at 1 month (panels a-c) and 3 months (panels d-f) after instillation at different magnifications. At any of the time points, SWCNTs deposited in the lungs were typically phagocytosed by alveolar macrophages or macrophages in the interstitial tissues.

On the basis of TEM observation, SWCNTs deposited in the lungs were observed as they were phagocytosed by alveolar macrophages or macrophages in the interstitial tissues at any of the time points (Figure 11). SWCNTs in the lungs were presented in a form similar to the test solution. Individual SWCNTs in the cells of the interstitial tissue were not observed.

## Discussion

In the present study, the pulmonary and systemic responses of highly pure, well-dispersed, and well-characterized SWCNTs were examined after intratracheal instillation in rats. Exposure to SWCNTs up to 2 mg/kg did not produce mortality, changes in clinical signs, or body weights during the observation period. Pulmonary inflammatory responses based on the lung weight, BALF inflammatory cells, and biochemical parameters such as LDH value, protein contents, and IL-1β and IL-6 activities, and histopathological changes were observed dose-dependently. In the 0.04mg/kg SWCNT-exposed group, almost no changes were observed during the observation period. In the 0.2 mg/kg SWCNT-exposed group, pulmonary inflammatory responses were observed after instillation. In the 1 mg/kg and 2 mg/kg SWCNT-exposed group, enhancement of the pulmonary inflammation and subsequent granuloma accompanied by increased lung weights were observed. Furthermore, the histopatho-logical findings in the lungs of rats exposed to SWCNTs showed inflammatory responses related with the vital reaction to the foreign substance that was instilled intratracheally.

In all the groups, other cytokines (i.e. IL-1α, IL-2, IL-4, IL-10, GM-CSF, INF-γ, and TNF-α) and HO-1 levels in the BALF were not significantly increased. SWCNTs deposited in the lungs were observed, as they were phagocytosed by alveolar macrophages or macrophages in the interstitial tissues at any of the time points. Individual SWCNTs in the cells of the interstitial tissue were not observed. SWCNTs were not found in the other organs (i.e. the liver, kidney, spleen, and cerebrum) at any of the time points.

A small number of reports are available that assessed the pulmonary toxicity of SWCNTs. However, most of the previous studies were conducted with agglomerated SWCNTs or SWCNTs containing relatively high impurities.

The pulmonary responses of SWCNTs were examined after intratracheal instillation in rats, and transient inflammatory and cell injury effects were observed in 1 or 5 mg/ kg SWCNT-exposed rats at 3 months after instillation (Warheit et al., 2004). The major airways were mechanically blocked by the agglomerated SWCNTs at a dose of 5 mg/kg and led to suffocation in 15% of the CNT-exposed rats. Non-dose-dependent pulmonary responses due to instillation of SWCNTs were reported (Warheit et al., 2004), although the severity of the lesions on histopathological examination of rat lungs was dose dependent in the present study, suggesting that the dispersion state of SWCNTs is an important factor for SWCNT toxicity. Lam et al. (2004) examined the pulmonary effect of a single dose of SWCNT (17 mg/kg) after intratracheal instillation by an incision of ventral neck skin in mice. They reported that deaths occurred 4 to 7 days after instillation of the SWCNTs containing Ni and Y; however, no deaths occurred in animals exposed to two SWCNT products that contained iron; the authors attribute the death to Ni toxicity (Lam et al., 2004, 2006). Inflammatory responses and granuloma formation were also reported in their study. The pulmonary responses of SWCNTs were also examined after intratracheal instillation in mice (Chou et al., 2008). As a result, the pulmonary inflammatory response was observed in 0.5 mg SWCNT-exposed at 2 weeks after instillation. Shvedova et al. (2005) noted that pharyngeal aspiration of SWCNTs containing as much as 18% (w/w) iron in mice induced acute inflammation and interstitial fibrosis in the lungs, and suggested that the fibrotic response might differ from the mechanisms proposed for chronic activation of alveolar macrophages. Shvedova et al. (2007, 2008b, 2009) interpreted that the pulmonary responses induced by SWCNT exposure were enhanced in NADPH oxidase-deficient mice, vitamin E-deficient mice, and suggested that oxidative stress might participate in the pulmonary toxicity of CNTs. In the present study, pulmonary inflammatory responses were induced even at 3 or 6 months after instillation in 1 mg/kg and higher doses of SWCNT-exposed groups. Granulomas were also observed in the interstitium through observation periods in those groups of rats. Progressive lung tissue thickening responses were observed in the highest dose (2 mg/ kg) of SWCNT-exposed group. However, fibrosis, atypical lesion, or tumor-related findings were not observed in all groups up to 6 months after instillation. Purity of SWCNT samples, particularly metal impurity is also an important factor for SWCNT toxicity.

Some studies reported that surface area and particle number determinations appear to play important roles in facilitating ultrafine particle-related lung toxicity (e.g., Donaldson et al., 2001; Oberdörster et al., 2005). In the present study, pulmonary deposition amounts of SWCNTs were calculated in terms of the SWCNT mass, surface area, and particle numbers. The pulmonary deposition amount of SWCNTs in this study was considered to be the same as the instilled dose of the SWCNTs (i.e. 0.04, 0.2, 1.0, and 2.0 mg/kg). Based on the mean BET surface area of the bulk SWCNT samples (1064 m^2^/g), the doses can be expressed in terms of the CNT surface area dose, which are 0.04, 0.2, 1.0, and 2.0 m^2^/kg, for doses of 0.04, 0.2, 1.0, and 2.0 mg/kg, respectively. Furthermore, based on the length of the SWCNT samples per 1g (1.4 × 10^11^ m/g and assuming that the tube diameter and length are uniform (3.0 nm and 0.32 μm, respectively), and that SWCNTs dispersed in the suspension formed SWCNT bundles consisting of 10 individual tubes, the doses can also be expressed in terms of particle numbers, which are 1.8 × 10^12^, 8.8 × 10^12^, 4.4 × 10^13^, and 8.8 × 10^13^ particle/kg for doses of 0.04, 0.2, 1.0, and 2.0 mg/kg, respectively. In contrast, based on the BET surface area (5.0 m^2^/g), average diameter (1.7 μm), and density (2.65 g/cm^3^) of Min-U-Sil 5 crystalline silica particles, surface area and particle number dose in 5 mg/kg of crystalline silica-exposed group can be calculated as 0.025 m^2^/kg and 7.3 × 10^8^ particle/kg, respectively. Inflammatory responses induced by a relatively low dose (in terms of mass dose) of SWCNT exposure may be relevant to high surface area and high particle number doses.

We have also conducted an inhalation exposure study with the same SWCNTs in rats for 4 week (6 h/day, 5 day/ week) and then examined at 3 days, 1 week, and 3 months after exposure (Morimoto et al., submitted). Pulmonary deposition of SWCNTs in the inhalation study was estimated to be 0.014 and 0.06 mg/kg in groups of rats exposed to low (0.03 ± 0.003 mg/m^3^) and high (0.13 ± 0.03mg/m^3^) concentrations of SWCNTs. As a result, an adverse effect (i.e. inflammation) was not observed in either low or high concentration of SWCNT-exposed groups. Therefore, we have concluded that the 0.13mg/m^3^ of SWCNT concentration and 0.059 mg/kg of pulmonary deposition may correspond to no observed adverse effect level (NOAEL) for the acute period (Morimoto et al., submitted).

This result was consistent with our present intratracheal instillation study, assuming that pulmonary toxicity depends on the maximum deposition amount in lung. The deposited amount of SWCNTs in the 0.04mg/kg SWCNT-exposed group in the present study was almost the same as the high concentration-exposure group in the inhalation study (0.06mg/kg). In the 0.04mg/kg SWCNT-exposed group, inflammatory response was only observed at 3 days after instillation. In contrast, 0.2 mg/kg of SWCNT instillation exposure induced pulmonary inflammatory response after instillation, and a dose of more than 1 mg/kg of SWCNTs induced persistent pulmonary inflammation for up to at least 6 months after instillation. These results indicate that pulmonary inflammation was induced with increased pulmonary deposition of SWCNTs.

## Conclusions

Intratracheal instillation of highly pure and well-dispersed SWCNTs in rats induced inflammatory responses in the lungs in a dose-dependent manner (see Table 3). However, this inflammatory response was not induced in other tissues (i.e. the liver, kidney, spleen, and cerebrum). Progressive lung tissue thickening responses were observed in the highest dose (2 mg/kg) of SWCNT-exposed group. However, fibrosis, atypical lesion, or tumor-related findings were not observed in all groups up to 6 months after instillation. SWCNTs did not induce pulmonary inflammation at 0.04 mg/kg (corresponding to approximately 0.04 m^2^/kg or 2.2 × 10^12^ fiber/kg) of pulmonary deposition. The present study is applicable to judge a NOAEL of highly pure and well-dispersed SWCNTs.
